# Variants of papillary thyroid carcinoma: association with histopathological prognostic factors

**DOI:** 10.5935/1808-8694.20130135

**Published:** 2015-10-08

**Authors:** Fábio Muradás Girardi, Marinez Bizarro Barra, Cláudio Galleano Zettler

**Affiliations:** a Head and Neck Surgeon, member of the head and neck surgery team at Santa Rita Hospital, Irmandade Santa Casa de Misericórdia de Porto Alegre; b Pathologist; Department of Pathology, Santa Casa de Misericordia de Porto Alegre; c PhD in Pathology, Department of Pathology, Santa Casa de Misericordia de Porto Alegre. Irmandade Santa Casa de Misericórdia de Porto Alegre, Santa Rita Hospital

**Keywords:** papillary carcinoma, prognosis, thyroid neoplasms

## Abstract

Papillary carcinoma is the most common thyroid malignancy. Many variants of this tumor have been described, with different morphological and molecular characteristics. Although most cases have excellent prognosis, the relationship between tumor architecture and its biological behavior remains controversial.

**Objective:**

To present the experience of a single center on the prevalence of thyroid papillary carcinoma variants and their relationship with other histopathological prognostic factors.

**Method:**

Retrospective study of all the cases submitted to thyroidectomy for papillary carcinoma in the same institution over 11 years.

**Results:**

We included 517 patients, 81.9% of them were women. The average age was 47.2 years. The variants recognized to have higher aggressiveness potential corresponded to 5.6% of the sample. We found an association of tumor subtypes with greater lesion diameter, T staging, lymphovascular and gland capsule invasion.

**Conclusion:**

A small percentage of papillary carcinoma cases is represented by variants recognized by their greater potential for aggression. There are associations between these variants and several other histopathological factors already recognized for their prognostic value, which may, by themselves, influence the outcome of these cases.

## INTRODUCTION

Papillary carcinoma (PC) is the most common thyroid malignancy. Generally, it is an indolent disease and it has a good prognosis if completely resected. However, some cases usually related to some specific clinicopathological parameters may yield worse outcomes. The TNM classification is the most used in the risk classification of malignant tumors^1^. Lymph node metastasis in the lateral compartment (N1b), massive extrathyroidal disease (T4) and distant metastasis (M1) are independent factors that have been correlated with poor prognosis in these patients^2^.

Although some studies on the prognostic value of some PC variants have already been developed, our knowledge about the prevalence and biological behavior of these variants is still poor, especially due to sample size. In our study, we investigated the prevalence of these histological variants by means of a large institutional series, comparing clinicopathological characteristics according to histological subtype and between groups with variants recognized by their greater or lesser aggressiveness potential.

## METHOD

### Patients

The histopathological records of all patients who underwent thyroidectomy with final histopathological diagnosis of thyroid PC from June 2000 to December 2010 were reviewed at our institution. All patients underwent clinical and ultrasound evaluation preoperatively. Relevant cases underwent cytologic evaluation of thyroid nodules by Fine Needle Aspiration (FNA). The central or lateral compartment neck dissection is not performed electively in our institution, being reserved for patients with clinical or ultrasound suspicion of lymph node metastases. Many cases of PC, cases of poorly differentiated carcinoma, tumors with undifferentiated zones, cases without description of the size of the largest nodule, PC cases with focal insular component, as well as medullary neoplasia associated with thyroid PC, were taken off the study.

The following parameters were entered into a specific database (Microsoft® Excel 2003 version, Microsoft Corporation, Redmond, WA, USA): age, gender, concomitant Hashimoto's thyroiditis, associated lymph node dissection, detailed histopathological description with information on the tumor variant or tumor differentiation pattern, predominant nodule diameter, multifocality, extrathyroidal extension, lymph node group compromised and T and N staging. We did not evaluate distant metastasis, since only the histopathological reports alone were reviewed.

### Definitions and Pathology

Tumors were classified into each variant or differentiation pattern, similar to that of the World Health Organization (WHO) classification^3^ as: Follicular, Oncocytic, Clear Cells, Diffuse Sclerosing, Tall Cells, Solid, Cribriform, Fasciitis-like, Macrofollicular, and Microcarcinoma. There were no cases belonging to the macrofollicular and cribriform variants in our sample. Besides these, we added the Columnar and the Warthin-like differentiation pattern, present in six cases. The conventional, usual or typical thyroid PC cases were classified as belonging to the Classic pattern or variant. When evaluating the microcarcinomas, we ran descriptive analysis of tumor differentiation patterns similarly to the previously described variants. For statistical purposes, we grouped differentiation patterns and tumor variants. When more than one variant or differentiation pattern were present in the same case, these were considered complex variants or tumor patterns. These were described as shown on Table 1.

**Table 1:** Distribution of tumor patterns and variants among complex tumors.

TU > 1 cm (N = 29)	Tumor variants	N
	Follicular	20
	Classic	16
	Solid	15
	Oncocytic	8
	Tall cells	3
	Columnar	2
	Clear Cells	1
	Diffuse sclerosing	1
TU ≤ 1 cm (N = 7)	Differentiation pattern	
	Follicular	5
	Classic	4
	Oncocytic	3
	Solid	1

TU: Tumor; N: Absolute frequency; %: Relative frequency; cm: Centimeters.

Tumors were considered multifocal when two or more foci were found in one or both lobes. Hashimoto's thyroiditis has been suggested based on histopathological findings. Papillary microcarcinomas were defined as tumors with no more than 1.0 cm in diameter at the final histological examination. The Tall, Columnar, Solid or Diffuse Sclerosing Cells were defined as variants or differentiation patterns with greater aggressiveness potential; and those with the less malignancy potential were the Classical, Follicular and Oncocytic types. Other variants or patterns of tumor differentiation were excluded from the statistical calculation and are only presented descriptively.

Complex tumor cases with tumor nodules or zones classified in the higher malignancy category were grouped with other similar cases for the purpose of statistical calculation and are listed on Table 2. The same was true among cases with low malignancy potential. Pathological staging was performed according to the seventh edition of the American Joint Committee on Cancer pTNM staging system^4^. Lymph node status was defined by pathological evidence of metastases in the lymph nodes which were removed. Extraglandular involvement was defined based on evidence of tumor infiltration beyond the capsule gland upon microscopic examination. We performed a comparative analysis of clinical and histopathological variables among the Classic, Follicular and Oncocytic variants, and among the groups with variants recognized by the highest and lowest potential for aggression, as previously agreed. All the data was collected by the same researcher (Girardi FM) and the entire pathology review was carried out by the same pathologist (Barra MB).

**Table 2:** Descriptive analysis of the clinicopathological traits among variants or differentiation patterns carrying a higher malignancy potential.

Case	Gender	Age	Variant or differentiation pattern	Hashimoto	Multifocality	Lymph vascular/neural invasion	Largest nodule diameter	Gland capsular invasion	Extraglandular spill	T	N
1	F	34	Tall cells	A	A	A	2.6	P	A	T2	N0
2	F	53	Tall Cells + sclerosing (< 50%) + follicular (< 50%)	A	P	A	2	P	P	T3	N0
3	F	56	Tall Cells (< 50%) + follicular + oncocytic (< 50%)	P	P	A	2	A	A	T1	N0
4	F	68	Column	A	A	A	0.5	A	A	T1	N0
5	F	56	Column (< 50%) + solid (< 50%) + follicular	A	P	A	2.5	P	A	T2	N0
6	F	67	Column + solid (< 50%) + follicular (< 50%)	P	P	A	4	P	A	T2	N0
7	F	61	Sclerosing	A	A	A	7	P	A	T3	N0
8	F	35	Sclerosing	A	P	A	1.1	P	P	T3	N1b
9	F	25	Sclerosing	A	A	P	2.5	P	P	T3	N1b
10	F	39	Sclerosing	P	P	A	3	P	P	T3	N1b
11	F	68	Sclerosing	A	A	A	1	P	P	T3	N0
12	F	49	Solid	A	A	A	0.8	A	A	T1	N0
13	F	77	Solid	A	P	A	1.5	A	A	T1	N0
14	F	41	Solid	A	P	A	2	P	P	T3	N1a
15	F	60	Solid	A	A	A	5.5	A	A	T3	N0
16	M	64	Solid (< 50%) + Tall Cells (< 50%) - follicular	A	A	P	6.5	P	A	T3	N1b
17	M	69	Solid + clear cells (< 50%)	A	A	P	8	P	P	T3	N1b
18	F	63	Solid + classic (< 50%)	P	A	A	0.4	A	A	T1	N0
19	F	33	Solid (< 50%) + classic	A	A	A	5	P	P	T3	N0
20	M	72	Solid (< 50%) + classic	A	A	P	6.4	P	P	T3	N0
21	F	46	Solid + follicular (< 50%)	P	P	A	1.5	P	A	T1	N1a
22	F	43	Solid + follicular (< 50%)	A	A	A	3.8	A	A	T2	N0
23	M	29	Solid + follicular (< 50%)	A	A	A	3.6	P	A	T2	N0
24	F	23	Solid + follicular (< 50%)	A	A	P	4.5	P	P	T3	N0
25	F	51	Solid + follicular (< 50%) + classic (< 50%)	A	P	A	2	P	A	T1	N0
26	F	56	Solid (< 50%) + follicular + oncocytic (< 50%)	P	A	A	2.2	P	A	T2	N1a
27	F	58	Solid + follicular (< 50%) + oncocytic (< 50%)	A	P	A	1.8	P	P	T3	N0
28	F	38	Solid (< 50%) + oncocytic	A	A	A	1.2	P	P	T3	N0
29	F	13	Solid (< 50%) + oncocytic (< 50%) + classic	A	A	P	3.1	P	P	T3	N0

F: Females; M: Males; Age in years; Diameter in centimeters; A: Absent; P: Present; T: Staging T; N: Staging N.

### Statistics and Ethical Aspects

Descriptive analysis was used to summarize the data. We performed the Kolmogorov-Smirnov test to assess normality of continuous variables. Continuous variables with normal distribution were expressed as mean and standard deviation. Those with abnormal distribution were also expressed as median, minimum and maximum values. Categorical variables were expressed as absolute and relative frequency. We used *Student's t* test and ANOVA for comparing mean ages; the Mann-Whitney U test and Kruskal-Wallis tests were used for comparing tumor diameter and the nonparametric chi-square test was used to compare categorical variables. Statistical analysis was performed using the EpiInfo software, version 3.4.3, 2007. All tests considered significance level of 5%.

The authors guarantee data keeping and the confidentiality of the material obtained. As there were no interventions, we did not have to use the Informed Consent Form. The project was approved by the Ethics Committee of our institution (Project No 3483/11).

## RESULTS

Between June 2000 and December 2010, there were 623 thyroidectomy for thyroid cancer performed in our institution. Eight cases were excluded due to lack of tumor diameter information, all corresponding to Classic PC. Altogether, 517 (82.9%) patients met the inclusion criteria. Of the total, 81.9% were women. The male:female ratio was 1:4.5. The average age was 47.20 years, range of 13–87 years. We observed that the Microcarcinoma variant was the most prevalent, with 42.1% of cases, followed by the Classical and Follicular variants, respectively (Table 3).

**Table 3:** Descriptive analysis of tumor patterns and variants among cases of thyroid papillary carcinoma.

Variants or differentiation patterns	N (517)	% (100)
Classic variant	164	31.7
Follicular variant	83	16.0
Oncocytic variant	10	1.9
Diffuse sclerosing variant	4	0.7
Warthin-like variant	4	0.7
Solid variant	3	0.5
Tall Cells variant	1	0.1
Fasciite-like variant	1	0.1
Complex tumors	29	5.6
Microcarcinoma variant	218	42.1
Classic pattern	174	----
Follicular pattern	25	----
Oncocytic pattern	8	----
Solid pattern	1	----
Columnar pattern	1	----
Sclerosing diffuse pattern	1	----
Warthin-like pattern	1	----
Complex tumors	7	----

N: Absolute frequency; %: Relative frequency.

In 36 cases there were more than one tumor variant or differentiation pattern in the same gland, and the Classic variation or differentiation pattern was the most prevalent in those cases. Except for nodule size, we did not detect statistically significant differences between the Classical, Follicular and Oncocytic variants or differentiation pattern (Table 4). However, this difference was not maintained when we compared the diameter between the Classical and Oncocytic variants (*p* = 0.2842), as well as when we compared the Follicular and Oncocytic variants (*p* = 0.2129), only the Classical and Follicular variants, as per the Mann-Whitney U test *(p* = 0.002).

**Table 4:** Analysis of the clinicopathological characteristics among tumor variants or differentiation patterns (classic × follicular × oncocytic).

Variant/differentiation pattern									
	Classic		Follicular		Oncocytic		Total		*p*-value
	Mean (SD)		Mean (SD)		Mean (SD)		Mean (SD)		
Age	46.6 (14.00)		47.75 (13.94)		50.81 (12.99)		47.04 (13.95)		0.3785
Tumor diameter (m ± SD)	1.39 (1.29)		2.27 (1.74)		1.88 (1.61)		1.63 (1.48)		
	Median (Min-Max)		Median (Min-Max)		Median (Min-Max)		Median (Min-Max)		
Tumor diameter	1 (0.01–7.5)		1.6 (0.1–9)		1.3 (0.2–6)		1.2 (0.01–9)		0.0290
	N (329)	% (70.7)	N (118)	% (25.3)	N (18)	% (3.8)	N (465)	% (100)	
Men/Women	56/273	17.0/82.9	24/94	20.3/79.6	4/14	22.2/77.7	84/381	18.0/81.9	0.6490
Associated Hashimoto's thyroiditis	44	13.3	22	18.6	3	16.6	69	14.8	0.3756
Lymph-vascular/neural invasion	18	5.4	11	9.3	0	0	29	6.2	0.1783
Multifocality	117	35.5	44	37.2	4	22.2	165	35.4	0.4603
Extraglandular spill	96	29.1	30	25.4	6	33.3	132	28.3	0.6610
Gland capsular invasion	177	53.7	60	50.8	8	44.4	245	52.6	0.6656
Positive neck LN	73	22.1	25	21.1	4	22.2	102	21.9	0.9744
LNs involved in the lateral compartment	28	8.5	7	5.9	0	0	35	7.5	0.3082
T Stage: 1–2	226	68.6	78	66.1	12	66.6	316	67.9	0.8684
T Stage: 3–4	103	31.3	40	33.8	6	33.3	149	32.0	

N: Absolute frequency; %: Relative frequency; SD: Standard deviation; Min-Max: Variation between minimum and maximum; Age in years; Diameters in centimeters; The underscored diameter values differ among themselves as per the Mann-Whitney U test (*p* = 0.002); LNs: Lymph nodes. Notice: We excluded 17 cases of complex tumors, six of them with oncocytic zones; *p*-value: Level of significance utilized. N: Absolute frequency; %: Relative frequency; SD: Standard deviation; Min-Max: Variation between minimum and maximum; Age in years; Diameters in centimeters; The underscored diameter values differ among themselves as per the Mann-Whitney U test (*p* = 0.002); LNs: Lymph nodes. Notice: We excluded 17 cases of complex tumors, six of them with oncocytic zones; *p*-value: Level of significance utilized. N: Absolute frequency; %: Relative frequency; SD: Standard deviation; Min-Max: Variation between minimum and maximum; Age in years; Diameters in centimeters; The underscored diameter values differ among themselves as per the Mann-Whitney U test (*p* = 0.002); LNs: Lymph nodes. Notice: We excluded 17 cases of complex tumors, six of them with oncocytic zones; *p*-value: Level of significance utilized. N: Absolute frequency; %: Relative frequency; SD: Standard deviation; Min-Max: Variation between minimum and maximum; Age in years; Diameters in centimeters; The underscored diameter values differ among themselves as per the Mann-Whitney U test (*p* = 0.002); LNs: Lymph nodes. Notice: We excluded 17 cases of complex tumors, six of them with oncocytic zones; *p*-value: Level of significance utilized.

As noted on Tables 2 and 5, those variants recognized by having the highest malignancy potential accounted for only 5.6% of the sample, and the solid variant predominated. We did not observe statistically significant differences between age, gender, multifocality, Hashimoto's thyroiditis and positive neck lymph nodes between groups with lower or higher malignancy potential variants or differentiation pattern (Table 5). However, we observed their association with larger tumor size, lymphovascular and gland capsule invasion and T staging.

**Table 5:** Analysis of the clinicopathological traits according to tumor variants or differentiation pattern (high × low malignant potential).

Variant/differentiation pattern							*p*-value
	High malignancy potential		Low malignancy potential		Total		
	Mean (SD)		Mean (SD)		Mean (SD)		
Age	49.89 (16.50)		47.04 (13.96)		47.20 (14.11)		0.3685
Tumor diameter	3.03 (2.06)		1.64 (1.48)		1.72 (1.55)		
	Median (Min-Max)		Median (Min-Max)		Median (Min-Max)		
Tumor diameter	2.5 (0.4–8)		1.2 (0.01–9)		1.2 (0.01–9)		0.0229
	N (29)	% (5.6)	N (482)	% (93.9)	N (511)	% (100)	
Men/Women	4/25	13.7/86.2	88/394	18.2/81.7	92/419	18.0/81.9	0.7197
Associated Hashimoto's thyroiditis	4	12.9	69	14.3	73	14.2	0.8273
Lympho-vascular/neural invasion	6	20.6	30	6.2	36	7.0	0.0098
Multifocality	11	37.9	183	37.9	194	37.9	0.9969
Extraglandular spill	13	44.8	139	28.8	152	29.7	0.1052
Gland capsular invasion	22	75.8	257	53.3	279	54.5	0.0296
Positive neck LNs	8	27.5	107	22.1	115	22.5	0.6558
LNs involved in the lateral compartment	5	17.2	36	7.4	41	8.0	0.1261
T Stage: 1–2	13	44.8	325	67.4	338	66.1	
T Stage: 3–4	16	55.1	157	32.5	173	33.8	0.0217

N: Absolute frequency; %: Relative frequency; SD: Standard deviation; Min-Max: Variation between minimum and maximum; Age in years; Diameters in centimeters; LNs: Lymph nodes. Notice: We excluded 17 cases of Warthin-like and fasciite-like tumors; *p*-value: Level of significance utilized.

## DISCUSSION

The most prevalent variant or differentiation pattern in our study, grouped microcarcinomas, was the Classic, followed by the Follicular variant or pattern. Together accounted for 86.2 % of the total. Similar percentages have been observed in other series5., 6.; and the study by Lam et al.^5^ was the one that most closely approximated to the figures in our sample. Studies have shown that, despite some histological differences observed between the Classic and Follicular variants of thyroid PC, both neoplasms have favorable prognosis and similar cancer-specific survival at 10 and 15 years^7^. Lang et al.^7^ observed fewer metastatic lymph nodes and lower extraglandular overflow rate among the Follicular variant cases when compared to the usual forms of PC. Ozdemir et al.^8^ showed greater tumor diameter, although lower prevalence of capsular invasion and extraglandular extravasation among Follicular variant cases when compared to the Classic one.

Likewise, although there are differences in the literature^9^, in most cases involving the Oncocytic variant of thyroid PC, the tumor is confined to the gland without evidence of association with poor prognosis histological features^10^. There are molecular differences between Oncocytic Carcinomas and the Oncocytic variant of the thyroid PC, suggesting that both diseases have different are genetic behavior^11^. Moreover, under the same staging classification, there is no evidence that the thyroid PC Oncocytic variant differs from the usual forms and the PC Follicular variant in biological behavior and malignancy potential6., 10..

In our study, except for tumor size, neither difference was observed between the clinical and pathological parameters involving the variants: Classic, Follicular and Oncocytic. In the analysis of subgroups, even diameter differences do not sustain, this being a different characteristic among the cases of Follicular and Classic variants in the sample, similar to the study by Ozdemir et al.^8^. It is known that the follicular variant diagnosis of CP in the preoperative period is a challenge, both from the cytology point-of-view^12^ as well as the clinical-echographical point of view^8^, which may have delayed the diagnosis of this tumor variant and justify the larger diameter this histological subtype in relation to the classical form of PCin our sample.

Many different histologic variants have been described for the thyroid PC. Some variants have been associated with worse prognosis^13^. Michels et al.^14^ developed a study analyzing survival between patients with regular thyroid PC and those with the Tall Cells variant. The 10-year survival was 90% and 79%, respectively. In an univariate analysis, the Tall Cells variant was associated with worse outcomes, which was not confirmed in the multivariate analysis. Other authors have developed similar studies involving other histological subtypes recognized by their worst prognosis, and in all of them, the evidence that the histological variant is an independent predictor with respect to outcome is weak^13^. The Columnar variant, similarly to the Tall Cells variant, shows an association with advanced locoregional disease and distant metastases^6^. Falvo et al.^15^ developed a comparative study of 83 cases of Diffuse Sclerosing variant with 183 cases of the usual forms. They concluded that the Diffuse Sclerosing variant is characterized by intrathyroidal spread and a high rate of lymph node and lung metastases. Sywak et al.^16^ reported that the Solid variant has a high propensity to extrathyroidal extension and lymph node metastasis. Like the previously described studies, we observed association between variants or cases with differentiation pattern having greater potential for malignancy and various histological features historically known for their association with poor prognosis.

Despite the representative sample volume of our study, cases recognized by worst potential prognosis are rare and account for a small part of the total number of thyroid PC cases, which makes it difficult to compare the clinical and histopathological data between each variant separately. Jung et al.^17^ in an institutional series of 14 years, gathered 23 cases of variants with the greatest malignancy potential (ten cases of the Tall Cell variant, five Diffuse Sclerosing, four Columnar, three of the Solid variant and one mixed case between the Columnar and Tall Cell variants) for comparative analysis of prognostic factors and outcomes in a group of cases of poorly differentiated thyroid carcinoma. Similarly, we chose to unify all cases of variants historically known for their association with poor prognosis in a single comparison group. Little is known about the role played by worse prognosis variants' foci or zones in the context of Classic, Follicular or Oncocytic variants or patterns. We chose to include them with the other cases with the greatest potential for malignancy for statistical calculation purposes. All cases that contained variants or patterns of differentiation recognized by worse prognosis are individually listed on Table 2, with discrimination of the predominant subtype, which we believe can collaborate in number for subsequent studies from other research centers, given the low incidence of these tumor variants even in large series.

There is a hypothesis that the thyroid PC has its beginnings in the Follicular Classic pattern, and with time it transforms into more malignant forms, depending on some molecular events, all the way to poorly differentiated and anaplastic tumors. Thus, it is postulated that patients with more malignant forms should be older, acquiring, in the course of the disease, all the histopathological characteristics already recognized of poor prognosis^18^. This model has been well studied among Tall Cell variants, represented in our study by only four cases with ages ranging between 34–64 years. In our study, we found no statistical correlation of age with tumor variant, which we believe may have been influenced by the low representation of this variant in our sample, besides excluding cases with areas of poorly differentiated carcinoma and tumors with areas of anaplasia.

## CONCLUSION

The Columnar, Tall Cells, Diffuse Sclerosing, Solid variants are usually more malignant than the Classical, Follicular and Oncocytic variants of the thyroid PC. Although prognostic studies using multivariate analysis, have publicized that the histologic subtype has lost strength as an independent predictor of worse outcomes, the forms for higher malignancy potential tend to be associated with several other factors historically reported in cases of unfavorable outcomes, alerting the physician that he/she is facing a potentially malignant tumor. We believe that our study came to add to the current knowledge, especially as a substrate for further studies involving more than one research center, given the low occurrence of some of these variants.
